# Microorganisms in coastal wetland sediments: a review on microbial community structure, functional gene, and environmental potential

**DOI:** 10.3389/fmicb.2023.1163896

**Published:** 2023-06-02

**Authors:** Shen Liang, Huai Li, Haitao Wu, Baixing Yan, Aiwen Song

**Affiliations:** ^1^Key Laboratory of Wetland Ecology and Environment, Northeast Institute of Geography and Agroecology, Chinese Academy of Sciences, Changchun, China; ^2^University of Chinese Academy of Sciences, Beijing, China

**Keywords:** coastal wetlands, microorganisms, community structure, functional gene, environmental potential

## Abstract

Coastal wetlands (CW) are the junction of the terrestrial and marine ecosystems and have special ecological compositions and functions, which are important for maintaining biogeochemical cycles. Microorganisms inhabiting in sediments play key roles in the material cycle of CW. Due to the variable environment of CW and the fact that most CW are affected by human activities and climate change, CW are severely degraded. In-depth understanding of the community structure, function, and environmental potential of microorganisms in CW sediments is essential for wetland restoration and function enhancement. Therefore, this paper summarizes microbial community structure and its influencing factors, discusses the change patterns of microbial functional genes, reveals the potential environmental functions of microorganisms, and further proposes future prospects about CW studies. These results provide some important references for promoting the application of microorganisms in material cycling and pollution remediation of CW.

## 1. Introduction

CW are the transitional regions between the terrestrial and marine ecosystems, mainly including shallow seas, estuaries, mangroves, salt marshes, deltas, etc. CW have the vegetated zones (mangroves, salt marshes, and seagrass beds) and non-vegetated zones (mudflats and sandy beaches), which are critical areas connecting land, freshwater habitats, and the ocean (Levin et al., 2001). CW can provide many facilities for human activities such as fishing and breeding (Zhang and Shao, 2013), and also protect coastal zones in flooding (Narayan et al., 2017). Moreover, CW richen in biodiversity, material cycling, energy flow, and species migration and evolution, with high primary productivity (Cui et al., 2016). CW are the most vulnerable ecosystems due to ocean dynamics, river disturbance, and human activities (Wang et al., 2022), and its degradation (such as biodiversity decline, ecosystem function loss, and coastal vegetation reduction) may lead to biological invasions, water quality deterioration, and reduced coastal protection from flooding and storm events (Barbier et al., 2011).

Microorganisms are an important component of wetland ecosystems and play key roles in biogeochemical cycles (DeLong et al., 2006). Microbial community structure has significant differences depending on different soil properties and vegetation types. Soil properties and plant types are the main determinants of microbial community structure (Yu et al., 2014). The interdependence between plants and microorganisms has a critical role in regulating ecosystem services such as nutrient cycling, productivity, and pollutants degradation (Abdu et al., 2017). Microorganisms can decompose soil organic matter, promote sulfate reduction, sulfide/sulfur oxidation, iron reduction, nitrification, pollutant degradation, and help improve soil structure and enhance ecosystem stability (Behera et al., 2017). CW exhibit strong nutrient and salinity gradients due to freshwater and seawater interactions, affecting soil microbial composition. Furthermore, these changes in microorganisms can lead to the variations of the function and structure of CW (Webster et al., 2015).

**Figure 1:**
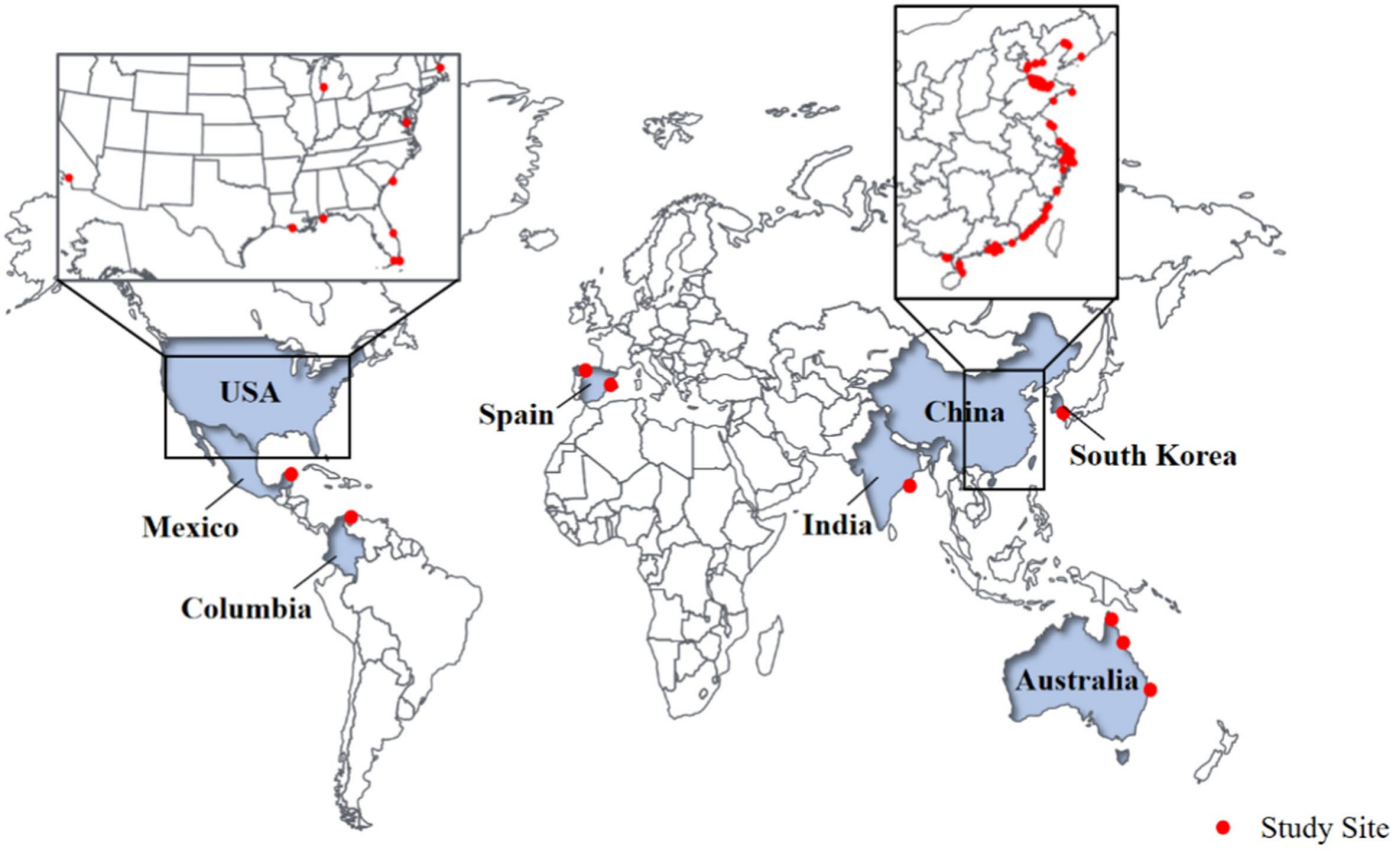
Location of CW studied by various groups for their microbial distribution.

Despite the importance of microorganisms in CW (Figure 1), few studies have reviewed and summarized them. Therefore, the purpose of this study is: (1) to provide an overview of microorganisms in CW sediments; and (2) to identify future research directions and possible difficulties. In this paper, we summarize the community structure characteristics, functional genetic variation and potential environmental functions of microorganisms in CW.

## 2. Microbial community structure in CW

The special soil characteristics and hydrological conditions of CW constitute a unique microbial community (Peralta et al., 2010). Linking microbial communities to physical, chemical, and biological factors can explore the drivers of microbial community formation (Fierer and Jackson, 2006), which is important for the restoration of environmental functions in wetland ecosystems.

### 2.1. Microbial community composition

Microbial species are abundant in CW, which are mainly divided into bacteria, archaea, and fungi (Table 1). Among them, bacterial communities have the highest richness, followed by archaeal and fungal communities (Cheung et al., 2018). Although the composition is the same at community level, there is some variation in microbial composition among different CW and different times of wetlands (Adame et al., 2012).

**Table 1:** The dominant microbial phylum in CW.

Location	Bacteria	Archaea	Fungi	References
**Asia**				
Yellow River Delta, China	Proteobacteria, Chloroflexi, Bacteroidetes, Actinobacteria, Gemmatimonadetes	Thaumarchaeota, Crenarchaeota, Euryarchaeota， Diapherotrites		Yu et al. (2012), Zhao et al. (2020), Lu et al. (2021) and Zhang et al. (2021)
Futian Mangrove Natural Reserve，China	Proteobacteria, Bacteroidetes, Firmicutes, Tenericutes, Chloroflexi			Tong et al. (2021)
Mai Po wetland, Hong Kong	Proteobacteria, Bacteroidetes, Chloroflexi, Gemmatimonadetes, Acidobacteria	Aenigmarchaeota, Bathyarchaeota， Euryarchaeota, Thaumarchaeota	Ascomycota, Basidiomycota, Chytridiomycota	Cheung et al. (2018)
Hangu District of Tianjin Municipality, China	Proteobacteria, Firmicutes, Bacteroidetes, Actinobacteria, Chloroflexi			Li et al. (2016)
Senmao farm in Rudong county, China	Bacteroidetes, Proteobacteria, Chloroflexi, Actinobacteria, Acidobacteria,			Bai et al. (2019)
Jiulong River Estuary, China	Bacteroidetes, Chlorobi, Chloroflexi, Proteobacteria, Firmicutes			Su et al. (2016)
Nalabana Island, India	Proteobacteria, Actinobacteria, Acidobacteria, Chloroflexi, Bacteroidetes	Euryarchaeota, Candidatus Bathyarchaeota, Thaumarchaeota, Crenarchaeota, Candidatus	Ascomycota, Basidiomycota, Mucoromycota, Chytridiomycota	Mohapatra et al. (2021)
**America**				
Eastern Coast of Florida, United States	Proteobacteria, Chloroflexi, Acidobacteria Nitrospirota, Chlorobi			Barreto et al. (2018)
York River State Park， United States	Proteobacteria, Acidobacteriota, Desulfobacterota, Bacteroidota, Chloroflexi	Halobacterota, Thermoplasmatota, Nanoarchaeota		Morina and Franklin (2022)
Whitney Marine Laboratory, United States	Proteobacteria, Bacteroidetes, Actinobacteria, Firmicutes, Cyanobacteria			Ward et al. (2019)
Barataria Bay, United States	Proteobacteria, Bacteroidetes, Firmicutes, Chloroflexi, Acidobacteria,			Bae et al. (2018)
Mangrove in La Guajira, Colombia	Proteobacteria, Actinobacteria, Chloroflexi, Bacteroidetes, Firmicutes,			Torres et al. (2019)
Point Aux Pins peninsula, United States	Proteobacteria, Firmicutes, Actinobacteria, Bacteroidetes, Acidobacteria			Beazley et al. (2012)
**Australia**				
East Trinity, Cairns, Australia	Proteobacteria， Chloroflexi, Bacteroidetes, Firmicutes, Acidobacteria	Crenarchaeota, Euryarchaeota		Ling et al. (2015)

Column “bacteria” selected the five phyla with the highest relative abundance.

Proteobacteria is the most abundant bacterial phyla in CW sediments (Ling et al., 2015), and mainly includes *α-, β-, γ-, δ-, and ε-Proteobacteria* (Hu et al., 2014), and their composition varies somewhat in different wetland types. For example, *γ-Proteobacteria* dominates in coastal zone of Yellow River Delta, whereas *γ-* and *δ-Proteobacteria* dominate in brine-freshwater zone (Hu et al., 2016). *ε-Proteobacteria* is dominant Proteobacteria in Jiuduansha Wetlands of Yangtze Estuary, while *γ- and β-Proteobacteria* are abundant in Jiangyanan Shoal of the river (Fei Xi et al., 2014). On the contrary, Firmicutes is the dominant phylum near salt flats of the Yangtze River Delta (Zou et al., 2020). In addition to Proteobacteria, Actinobacteria, Chloroflexi, Bacteroidetes, Firmicutes, Acidobacteria, and Planctomycetes are also the main phyla in CW (Zhang et al., 2017; An et al., 2019). Although bacterial compositions are relatively similar, there are some differences in different wetlands types. Moreover, archaea are also an important component of microbial communities and play an important role in biogeochemical cycles of CW (Narrowe et al., 2017). The dominant phyla of archaea are mainly Euryarchaeota, Thaumarchaeota, Bathyarchaeota and Grenarchaeota (Zhao et al., 2020; Chi et al., 2021a,b,c,d). Fungi as an important component of microorganisms and its community are essential for maintaining soil versatility (Li H. et al., 2019). Ascomycota and Basidiomycota are the dominant taxa in CW (Mohapatra et al., 2021). Among them, *Dothidomycetes* and *Sordariomycetes* are the dominant classes, and the dominant orders include *Pleosporales*, *Agaricales*, and *Capnodiales* (Ye et al., 2022). Moreover, many fungi cannot be attributed to the known phyla (Cheung et al., 2018).

### 2.2. Factors shaping microbial community

#### 2.2.1. Soil characterizations

**Figure 2:**
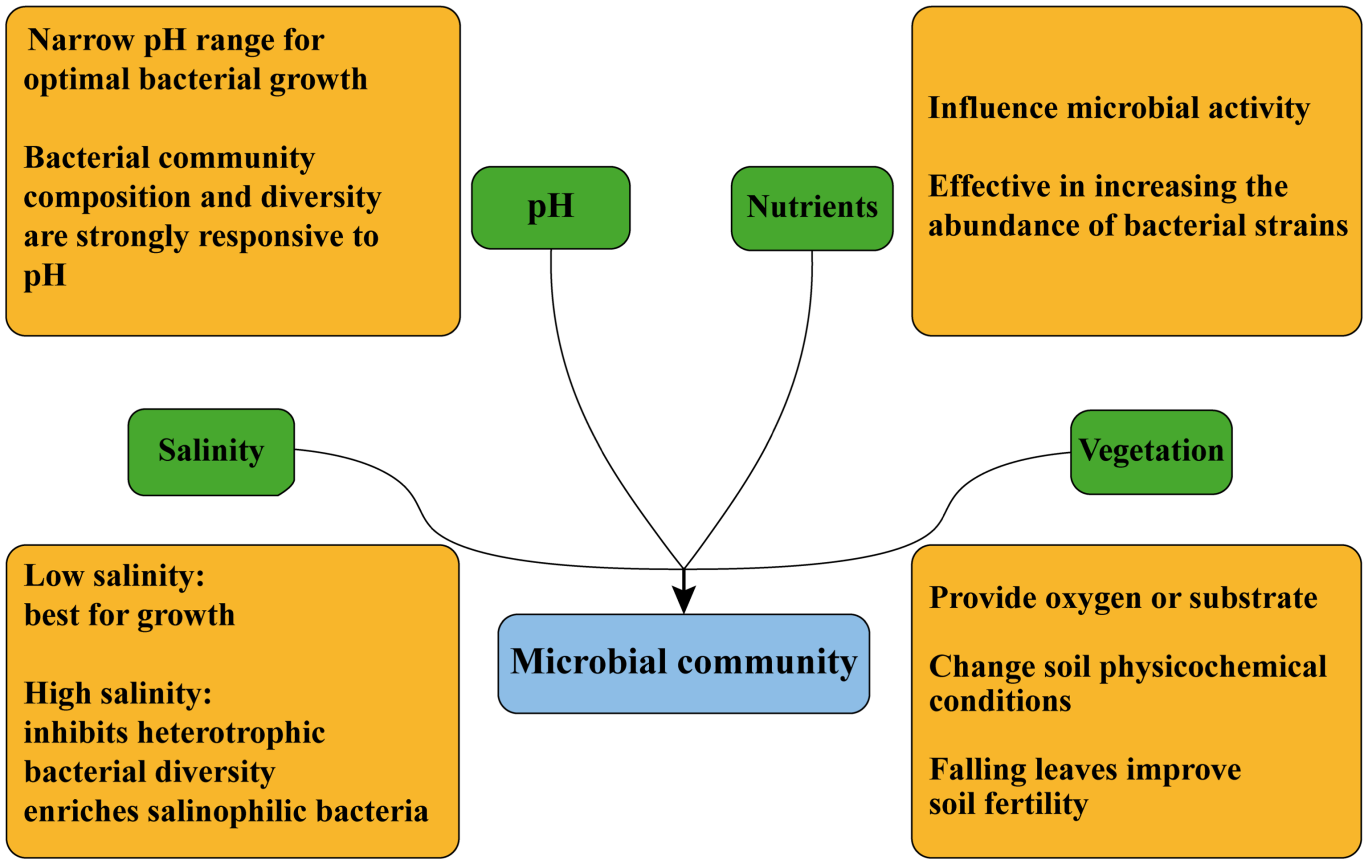
Factors influencing microbial community composition in CW.

Microorganisms in CW sediments are influenced by soil physicochemical properties, including salinity, pH, and nutrients (Jackson and Vallaire, 2009). These properties can affect microbial growth and metabolism as well as microbial activity (Figure 2).

Salinity can directly affect abiotic and biotic processes, and is considered as a major driver of ecosystem structure and function (Brucet et al., 2012). Previous study showed that salinity affected microbial communities and the associated biogeochemical cycles (Chambers et al., 2011). In general, salinity elevation usually has a negative impact on microorganisms, and low salinity environments are suited for microbial growth (Hu et al., 2016). High salinity can affect CW ecosystems through inhibiting plant growth and heterotrophic metabolism, and reducing soil quality and heterotrophic bacterial diversity (Abed et al., 2007). Microbial community structure varies along salinity gradients (Yang et al., 2018). It was found that halophilic bacteria such as *Fodinibius*, *Alkalilimnicola*, *Phycisphaera* and Gp21 were abundant in high-salinity zone of the Yangtze River Delta, and the dominant genera in the transition zone were *Rhodocyclus*, *Flavobacterium* and *Shin* (*Shinell*; Li J. et al., 2019).

pH has a significant effect on microbial community (Rousk et al., 2010). The bacterial composition and diversity in various ecosystems respond strongly to soil pH (Shen et al., 2013). Nitrospirae was lower in saline wetlands with high pH than in freshwater wetlands with low pH, and Nitrospirae was significantly negatively correlated with pH (Chi et al., 2021a,b,c,d).

Nutrients can also affect microbial growth. Total organic matter, total nitrogen and total phosphorus in the samples are usually tested as quantitative indicators of nutrient content when conducting experiments (Wang et al., 2012). The content of available nutrients affects microbial activity, and the addition of nutrients can effectively increase the abundance of bacterial strains (Meng et al., 2016). The unique structural composition of microbial communities in intertidal sediments of the Yellow River Delta is nutrient-related, and many saprophytic microorganisms are enriched (Zhang et al., 2017).

#### 2.2.2. Vegetation types

Vegetation has important influences on microbial community. Plants can create a unique environment for rhizosphere microorganisms (Grayston et al., 1998). Studies have shown that nutrient acquisition strategies of plant can drive the structural and functional formation of soil surface microbes and that changes in vegetation lead to changes in soil microbial diversity and function (Bahram et al., 2020). Root-mediated changes in soil can provide oxygen or other substrates for soil microbes (Noll et al., 2005) and also alter microbial community (Lipson et al., 2015). Plants alter the physicochemical conditions of sub-canopy soils (Menon et al., 2013), such as leaf litter can improve soil fertility and plant roots can release a variety of compounds into the surrounding soils (Garbeva et al., 2004). Roots of woody plants, such as mangroves, have different chemical (Perry and Mendelssohn, 2009) and physiological properties (Skelton and Allaway, 1996), and can transport different root secretions (Bertin et al., 2003). Differences of microbial community composition in mangrove- and swamp-dominated soils in Florida may be due to differences in root secretions or oxygen availability between vegetation types (Barreto et al., 2018). The photosynthesis of plants lead to the adsorption of cyanobacteria on plant rhizomes with more than 50% abundance in Yellow River Delta (Li et al., 2021).

## 3. Microbial functional genes in CW

### 3.1. Nitrogen cycle-related genes

**Figure 3:**
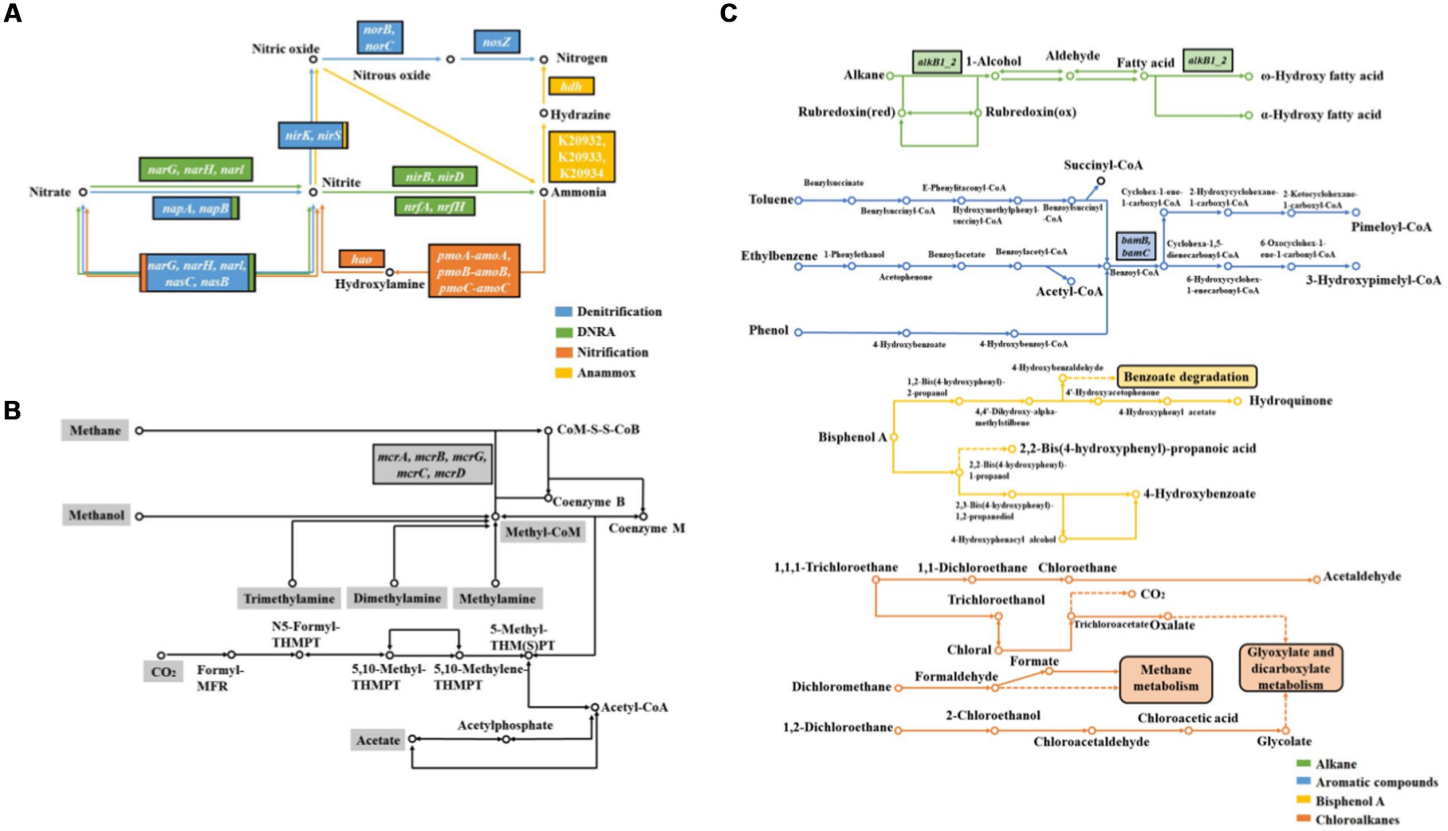
KEGG pathway of nitrogen cycle **(A)**, methanogenesis **(B)** and organics degradation **(C)** in CW.

Microbially mediated nitrogen cycle is one of the important components of biogeochemical cycles in CW (see Figure 3A). Among them, denitrification and dissimilatory nitrate reduction to ammonia (DNRA) processes are particularly important, and the end-products of these pathways have different effects on ecosystem nitrogen effectiveness (Morina and Franklin, 2022).

Denitrification plays a key role in nitrogen removal (van Breemen et al., 2002), and there are some important metabolic enzymes, including nitrate reductase (Nar), nitrite reductase (Nir), nitric oxide reductase (Nor), and nitrous oxide reductase (Nos; Levy-Booth et al., 2014). Among these, Nir catalyzes the rate-limiting step in denitrification, encoding by the *nirK* and/or *nirS* genes. A previous study found that the enzyme genes associated with denitrification decreased with increasing distance from the river bank of Yellow River, and reached their highest levels at distances of 0–50 m (except 0 m; Li W. et al., 2019). Saline plants had no significant effect on the abundances of denitrification genes *nirK*, *nirS*, and *nosZ* in Suncheon Bay, South Korea (Chaudhary et al., 2018). Mesosaline soils affect negatively on *nirS* and *nirK* genes compared to freshwater soils in the east coast of the United States (Morina and Franklin, 2022). Moreover, the diversity of *nirS* genes in Chinese CW exhibited significant latitudinal heterogeneity, and it is speculated that temperature rather than salinity contributes significantly to the latitudinal distribution of *nirS*-based denitrifying bacteria (Gao et al., 2016).

DNRA can convert nitrate nitrogen to ammonia nitrogen, and is one of the potentially important nitrogen cycling processes in CW. The reduction of NO_2_^−^ to NH_4_^+^ in DNRA is catalyzed by nitrite reductase, encoding by the *nrfA* gene. The abundance of *nirS* denitrifying bacteria is much greater than that of *nrfA*-DNRA microorganisms in the Chesapeake Bay watershed of United States, suggesting that denitrification is the primary nitrate reduction process (Franklin et al., 2017). The abundance of *nrfA* genes was low in tidal freshwater marshes in South Carolina of United States, suggesting weak DRNA process (Minick et al., 2019).

Nitrification and anammox are also important processes in the nitrogen cycle (Kuypers et al., 2018), but these two processes are weakly in CW ecosystems (Zhang X. et al., 2019). Functional genes associated with nitrification are mainly genes encoding ammonia monooxygenase (*amoA/B/C*) and hydroxylamine dehydrogenase (*hao*; Mohapatra et al., 2021), while those associated with anammox are mainly genes encoding hydrazine synthase (*hszA*; Harhangi et al., 2012). The abundance of functional genes associated with nitrification in intertidal wetlands disturbed by crabs is increased compared to the surrounding undisturbed sediments (An et al., 2021). Moreover, the copy number of nitrification genes is significantly higher in oily marshes (Bae et al., 2018).

### 3.2. Methanogenesis-related genes

Methane production is a major process in anaerobic carbon-cycle of CW, and methanogenic bacteria are the main microorganisms involved in this process, encoding by *mcrA* gene (see Figure 3B; Oremland et al., 1982). A previous study showed that the abundance of methanogenic genes in wetlands affected by runoff and tidal seawater increased with distance from the river bank, while gene abundance in tidal wetlands increased first and then decreased in Yellow River Delta (Chi et al., 2021a,b,c,d). The abundance of *mcrA* was significantly lower in oiled marshes compared to non-oiled marshes along the United States coast (Bae et al., 2018).

### 3.3. Organics degradation-related genes

A large number of organic pollutants from human activities are released into wetlands with industrial development, adversely affecting the surrounding ecosystems (Qian et al., 2016). Petroleum hydrocarbons are the main pollutants that affect the material cycle and ecosystem function of wetlands (Yuan et al., 2014). Indigenous microorganisms in wetlands can degrade petroleum hydrocarbons, and lots of hydrocarbon-degrading bacteria isolated from petroleum-contaminated soils play key roles in petroleum hydrocarbons degradation (Tiralerdpanich et al., 2018). Therefore, the level of petroleum hydrocarbon contamination in different wetland soils can affect microbial community, which leads to changes in metabolic functions (see Figure 3C).

Both *alkB* and CYP 153A1 genes encoding alkane hydroxylases are enriched in tidal marshs from the Coacheco River in the United States under chronically contaminated petroleum hydrocarbons such as gasoline, n-hexane, and dodecane (Ní Chadhain et al., 2018). The gene *alkB* involving in aerobic alkanes degradation has high copy number in oil-bearing coastal salt marshes of the United States, whereas *bamA* related to anaerobic aromatics degradation has low copy number (Bae et al., 2018). Genes associated with the degradation of alkanes, cycloalkanes, aromatic carboxylic acids, chlorinated aromatics, polycyclic aromatic hydrocarbons, and other aromatic hydrocarbons are significantly reduced in salt marshes of Gulf Coast during oil concentration reduction (Beazley et al., 2012). The initial dioxygenase and open-loop dioxygenase associated with phenanthrene (PHE) degradation were expressed under PHE contamination in CW, indicating the presence of aerobic PHE degradation (Chi et al., 2021a,b,c,d).

## 4. Environmental potential of microorganisms in CW

**Figure 4:**
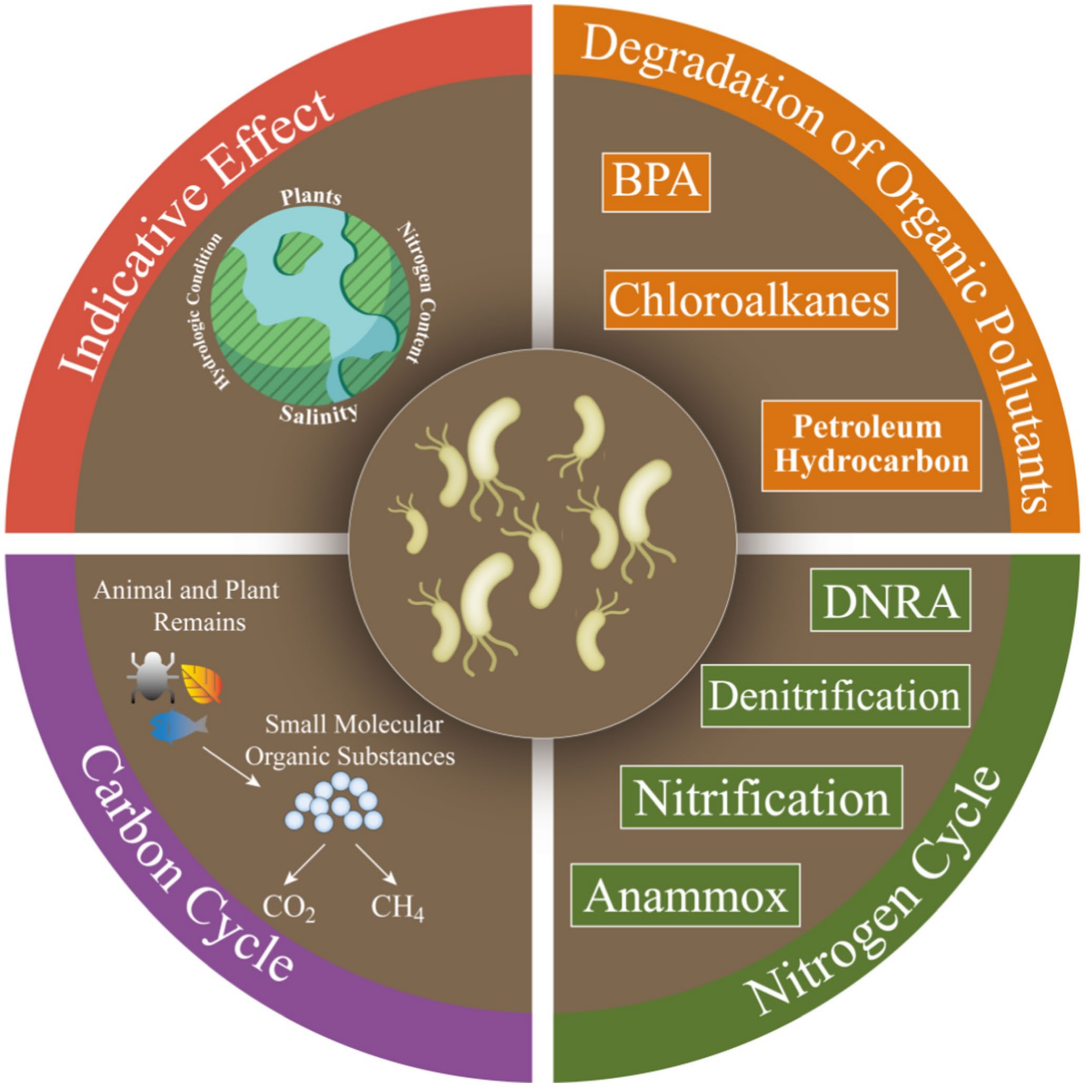
Potential environmental functions of microorganisms in CW.

Microorganisms contribute significantly to ecological functions (e.g., carbon and nitrogen cycle processes) in CW (Figure 4), which are critical in retaining chemical contaminants (e.g., organic pollutants) and excess nutrients (Horton et al., 2019).

### 4.1. Functional indicator

Previous studies have shown that the species composition and spatio-temporal dynamics of soil microbial communities are related to habitat characteristics, plant types, and human interferences (Eddie et al., 2010). Microorganisms are highly sensitive to environmental changes and thus can be an ideal indicator for environment monitoring (Santos et al., 2010; Yang et al., 2012). The indicative effects of microbial communities are various (Table 2). Fungal community composition in different habitats varies and could be used as a bioindicator to assess the restoration process of mangrove ecosystems in Jiulongjiang estuary (Yu et al., 2014). Fungi as indicator species of *P. australis* soils is found in restoration area of the Yangtze River Delta (Ma et al., 2017). Differences in microbial community over a short period in Florida suggest that they can serve as early warning signals for sea-level rise (Chambers et al., 2016). The ratio of ammonia to nitrate nitrogen in CW of Pearl River Delta significantly affect bacterial community composition, and thus anaerobic ammonia-oxidizing bacteria is a bioindicator of terrestrial nitrogen input or pollution (Han and Gu, 2015).

**Table 2:** Summary of indicative effects of microbial communities in CW.

Location	CW	Indicative effect	References
Jiulong River Estuary, China	M	Microbial community structure could be bioindicator of the mangrove recovery	Yu et al. (2014)
Yellow River Delta, China	P	Fungi could be bioindicator for soils under *P. australis*	Ma et al. (2017)
Pearl River Delta, China	M	Anammox bacteria community structures could be bioindicator of the anthropogenic/terrestrial inputs	Han and Gu (2015)
Bohai Economic Rim, China	P	Functional genes could be bioindicator of denitrification potential	Zhang X. et al. (2019) and Zhang Y. et al. (2019)
Quangang District, China	M	The genera *Mangrovibacterium* and *Mangrovimonas* can both be potential bioindicators of wetland restoration	Lin et al. (2021)
*Avicennia germinans*, Columbia	M	Firmicutes, Chloroflexi, Cyanobacteria and Gemmatimonadetes may be bioindicators of anthropogenic pollution	Torres et al. (2019)

Column “CW” defines the vegetation type in coastal wetlands: *Mangrove* (M) and *Phragmites australis* (P).

### 4.2. Organic pollutant degradation

Microorganisms have become popularly alternatives for pollutant bioremediation because they are environmentally friendly and cost-effective (Macaulay, 2015). Hydrocarbon-degrading microorganisms are ubiquitous in many environments (Head et al., 2006). Most studies on microbial hydrocarbon degradation have focused on environments highly exposed to hydrocarbons, such as areas surrounding oil deposits and hydrocarbon spills. *Pseudomonas*-type alkane-degrading bacteria are enriched in marshes nearby oil contamination, suggesting that oil degradation is important at this zone (Ní Chadhain et al., 2018). Bacterial community in sediments of Mexican coastal zone can degrade toluene, naphthalene, chloroalkanes, and chlorinated alkanes, but has low removals of aromatics, fluorobenzoates, and xylenes (Reyes-Sosa et al., 2018). *Proteobacteria* is responsible for the degradation of some phenolic compounds including bisphenol A (BPA) in mangrove of Shenzhen, and shows significant variation with BPA biodegradation (Tong et al., 2021). The correlation between fungal abundance and phenol oxidase activities in the Mai Po wetlands of Hong Kong suggests that fungi can contribute to soluble phenols reduction (Luo et al., 2018). The relative abundance of hydrocarbon-degrading bacteria (*Proteobacteria*, *Actinobacteria*, and *Bacteroidetes*) in hydrocarbon-contaminated sediments increases in salt marshes along the Gulf of Mexico (Beazley et al., 2012). PAHs-degrading bacteria (*Proteobacteria*, *Bacteroidetes*, *Firmicutes*, and *Chloroflexi*) in mangrove increase under polycyclic aromatic hydrocarbons contamination in the Jiulongjiang estuary (Su et al., 2016).

### 4.3. Biogeochemical cycles

Functionally diverse microbial communities in CW contribute to the biogeochemical transformation of elements such as carbon (C) and nitrogen (N; Yuan et al., 2014; Yang et al., 2022), and these biological processes mainly include carbon formation and degradation, carbon fixation and nitrogen metabolism, methane metabolism, and exogenous biodegradation and metabolism (Mohapatra et al., 2021).

*Halobacteria* and *Thaumarchaeota* are found in Yellow River Delta which can fix CO_2_ (Hu et al., 2016). Compared to mangrove, invasive *Spartina alterniflora* significantly can increase CH_4_ emissions and decrease CO_2_ emissions (Han and Gu, 2015). CH_4_ production was high in soils with saline plants in Suncheon Bay, Korea (Chaudhary et al., 2018). Nutrient transformation is related to highly active and adaptive bacterial metabolic channels in Chinese coastal zone (Zhang et al., 2022). When exposed to unstable substrates, microbial respiration is much higher and can produce more CO_2_ in mangrove and marsh soils along the east coast of Florida, United States (Barreto et al., 2018). In Liaohe River estuary of China, there is a positive correlation between soil respiration rates and *Clostridia* abundance, suggesting that anaerobic carbon decomposition is important in brackish wetland soils (Yang et al., 2018).

Changes in biomass and community structure may enhance soil N sequestration due to the abilities of special heterotrophic metabolism and refractory organics degradation of soil microorganisms (Tang et al., 2011). Denitrification is the main mechanism for nitrogen removal in CW of Bohai Sea (Zhang Y. et al., 2019), and ammonia-oxidizing archaea and bacteria are also important in global nitrogen cycle (Bai et al., 2019). Nitrate reduction rates are associated with denitrifying bacterial community in the protected area in Spain, suggesting that microbial communities are closely associated with N_2_O emissions (Bañeras and Ruiz-Rueda, 2012). Nitrogen and phosphorus additions can increase microbial denitrification in the absence of salinity (Chi et al., 2021a,b,c,d). Nitrification and denitrification rates are more higher in intertidal areas disturbed by crabs, and this process greatly contribute to N_2_O emissions (An et al., 2021).

## 5. Future prospects

Microorganisms in CW sediments are highly biodiverse and spatial heterogeneous, and play a key role in maintaining biogeochemical cycles. With the rapid development of molecular biology technologies, our understanding of microbial community and their potential functions has grown substantially. However, several major challenges remain in CW studies.

(1) Accurate identification of key factors affecting microbial community structure. Microorganisms in coastal wetlands are important for maintaining the normal biogeochemical cycle, so it is important to explore the factors affecting the community structure. The most studies about influencing factors focused on single factor, ignoring a combination of multiple factors. Therefore, it is important to elucidate the dominant factors affecting microbial community, which will help to maintain the stability of coastal ecosystems, prevent CW destruction, and restore degraded CW.

(2) In-depth reveal the potential functions of microorganisms, and decipher the relationship between functional stability and microbial biodiversity. Microorganisms are important for environmental management due to the community and functional diversities. The stable microbial community not only ensure the normal biogeochemical cycle, but also decompose complex pollutants into harmless substances through metabolic activities. Furthermpre, clarifying the coupling relationship between microbial diversity and functional stability and parsing the function of biological elements in habitat function ascension will help to maintain and improve the stability of CW function.

## 6. Conclusion

Microorganisms in CW have become important players involving in biogeochemical cycles and potential solutions for the treatment of difficult-to-degrade pollutants. Microbial community structure usually rapidly changes in response to environmental changes. Therefore, they can be used as indicators to detect changes in CW. Our studies discuss the changes in microbial composition of CW, summarize the effects of different factors on microbial community structure and the important functional genes, and further reveal the potential environmental functions of coastal microbes. Microbial communities involving in organic pollutant degradation and material cycling of CW have been well developed, but their functions and the relationship between functional stability and microbial biodiversity need to be further explored.

## Author contributions

SL: data curation and analysis and writing - original draft. HL: conceptualization, methodology, resources, supervision, writing - review and editing, project administration, and funding acquisition. HW, BY, and AS: conceptualization, methodology, data curation, and writing - review and editing. All authors contributed to the article and approved the submitted version.

## Funding

This work was financially supported by the National Key R&D Program of China (no. 2022YFF1300901), National Natural Science Foundation of China (no. 42077353), and Natural Science Foundation of Jilin Province (no. 20230101100JC).

## Conflict of interest

The authors declare that the research was conducted in the absence of any commercial or financial relationships that could be construed as a potential conflict of interest.

## Publisher’s note

All claims expressed in this article are solely those of the authors and do not necessarily represent those of their affiliated organizations, or those of the publisher, the editors and the reviewers. Any product that may be evaluated in this article, or claim that may be made by its manufacturer, is not guaranteed or endorsed by the publisher.
